# The UBC/SIRT5/DRP1 axis regulates mitochondrial dynamics to alleviate *Staphylococcus aureus*-induced oxidative stress and senescence in bovine mammary epithelial cells

**DOI:** 10.1371/journal.ppat.1013975

**Published:** 2026-02-12

**Authors:** Huijie Hu, Naiyuan Jiang, Juxiong Liu, Junlong Bi, Xuanting Liu, Bin Xu, Yu Cao, Wenjin Guo, Shoupeng Fu

**Affiliations:** 1 State Key Laboratory for Diagnosis and Treatment of Severe Zoonotic Infectious Diseases, Key Laboratory for Zoonosis Research of the Ministry of Education, Institute of Zoonosis, College of Veterinary Medicine, Jilin University, Changchun, China; 2 College of Animal Science and Technology, Jilin Agricultural University, Changchun, China; 3 Yunnan Province International Joint Research and Development Center for Veterinary Pharmaceuticals, Yunnan Agricultural University, Kunming, Yunnan, China; 4 Jilin Provincial Key Laboratory of Nutrition and Functional Food and College of Food Science and Engineering, Jilin University, Changchun, China; 5 College of Animal Science and Veterinary Medicine, Heilongjiang Bayi Agricultural University, Daqing, P. R. China; University of Bristol, UNITED KINGDOM OF GREAT BRITAIN AND NORTHERN IRELAND

## Abstract

*Staphylococcus aureus* (*S. aureus*)–driven senescence of bovine mammary epithelial cells is a key determinant of mammary gland health, yet its molecular basis remains poorly defined. Sirtuin 5 (SIRT5), a mitochondria-localized desuccinylase, may play an important regulatory role in this process. This study aimed to elucidate the mechanisms by which *S. aureus* drives cellular senescence and to define the contribution of the SIRT5–mitochondrial axis to delaying senescence. We found pronounced oxidative stress and cellular senescence in mammary tissues from cows with *S. aureus* mastitis, accompanied by marked downregulation of SIRT5. In an *S. aureus*-infected epithelial cell model, infection induced mitochondrial stress characterized by excessive mitochondrial fragmentation, loss of membrane potential, and increased mitochondrial superoxide, along with oxidative damage and cellular senescence. Mechanistically, *S. aureus* toxins and the toxin-induced inflammatory response cooperatively drove mitochondrial stress, which in turn increased intracellular bacterial burden and exacerbated cell death. During infection, SIRT5 protein abundance was significantly reduced. Mass spectrometry and co-immunoprecipitation analyses indicated that infection upregulated the ubiquitin-conjugating enzyme ubiquitin C (UBC), enhanced its interaction with SIRT5, and promoted ubiquitin-mediated degradation of SIRT5. Loss of SIRT5 increased succinylation of dynamin-related protein 1 (DRP1), inhibited its ubiquitin-mediated degradation, and led to its excessive accumulation on the outer mitochondrial membrane, thereby promoting excessive mitochondrial fission. Functionally, SIRT5 overexpression markedly alleviated mitochondrial stress, oxidative damage, and senescence phenotypes. When mitochondrial fission was forcibly enhanced, the cytoprotective effect of SIRT5 was substantially weakened, confirming that SIRT5 acts through a pathway dependent on mitochondrial integrity. Collectively, *S. aureus* infection releases toxins and induces inflammatory injury, during which UBC-mediated SIRT5 degradation activates DRP1-dependent mitochondrial hyper-fragmentation, aggravating mitochondrial stress, oxidative stress, and mammary epithelial cell senescence. These findings identify SIRT5 as a critical regulator of redox and mitochondrial homeostasis in mammary epithelial cells and a potential therapeutic target for mitigating oxidative damage associated with bovine mastitis.

## Author summary

*Staphylococcus aureus* (*S. aureus*) is a leading cause of bovine mastitis, an intramammary infection that reduces milk yield, increases antibiotic use, and results in substantial economic loss. One way this bacterium damages the udder is by driving bovine mammary epithelial cells into premature senescence, but how infection triggers this process has not been fully understood. Here, we show that *S. aureus* infection induces an inflammatory response via its secreted toxins, which in turn provokes mitochondrial stress. We identify the mitochondrial enzyme Sirtuin 5 (SIRT5) as a key defender in this context. During infection, the host ubiquitin-conjugating enzyme ubiquitin C (UBC) targets SIRT5 for degradation. Loss of SIRT5 leads to accumulation of the mitochondrial fission protein dynamin-related protein 1 (DRP1), causing excessive mitochondrial fragmentation, enhanced oxidative damage, increased intracellular bacterial burden, and aggravated cellular senescence. Restoring SIRT5 helps to preserve mitochondrial integrity and reduces oxidative injury and senescence; however, this protection is weakened when mitochondrial fission is forcibly maintained at a high level. Together, our findings reveal that the UBC–SIRT5–DRP1 pathway links *S. aureus* infection to mitochondrial damage and cellular senescence, and they highlight SIRT5 as a potential therapeutic target to protect the bovine mammary gland (S1 Fig).

## Introduction

Bovine mastitis is one of the most prevalent and economically devastating diseases in dairy herds, characterized by its recurrent nature and the challenges associated with prevention and control [1]. Current clinical treatment still primarily relies on antimicrobial agents, which are effective in eliminating pathogens and suppressing the progression of mastitis [2]. However, they lack efficacy in reversing mammary tissue damage—particularly oxidative stress triggered by inflammation. Among the various pathogens responsible for mastitis, *Staphylococcus aureus (S. aureus)* is a common and representative species [3]. It not only directly damages mammary epithelial cells but also induces the generation of reactive oxygen species (ROS) and activates oxidative stress responses, thereby exacerbating mammary injury [4]. Previous studies have demonstrated that inflammation and oxidative stress are major drivers of cellular senescence [5]. Mammary epithelial cells, as key effectors in milk synthesis and secretion, are highly susceptible to oxidative damage. The accumulation of senescent cells accelerates mammary functional decline, reduces milk yield, and significantly increases disease susceptibility, leading to higher mastitis recurrence [6]. Therefore, elucidating the molecular mechanisms of oxidative stress during mastitis and identifying effective antioxidative targets are of great importance for delaying mammary gland aging and reducing mastitis relapse.

Mitochondria are central organelles for cellular energy metabolism and play a decisive role in maintaining redox homeostasis [7]. Studies have shown that inflammation-induced oxidative stress can damage mitochondrial structures and disrupt mitochondrial function, forming a vicious cycle of damage and oxidative amplification [8,9]. As such, mitochondria represent a critical regulatory node for mitigating oxidative injury. Through dynamic processes of fusion and fission, mitochondria maintain structural integrity and functional balance. Excessive mitochondrial fission has been linked to ROS accumulation, calcium dysregulation, and apoptosis, whereas mitochondrial fusion facilitates the clearance of damaged mitochondria and restoration of metabolic equilibrium [10,11]. Hence, preserving mitochondrial fusion-fission balance is a promising strategy to alleviate oxidative stress-induced cellular damage.

Recent evidence suggests that proteins involved in mitochondrial dynamics are subject to various post-translational modifications [12,13]. In this context, the Sirtuin family was selected as the focus of this study. Sirtuins are NAD ⁺ -dependent deacylases broadly implicated in metabolic regulation and antioxidative stress responses [14]. Among them, Sirtuin 5 (SIRT5) is a mitochondria-localized isoform with minimal deacetylase activity but robust desuccinylase function, exerting unique biological effects in mitochondrial regulation and oxidative stress defense [15]. Studies have reported that SIRT5 enhances cellular ROS scavenging and attenuates oxidative injury by desuccinylating and activating isocitrate dehydrogenase 2, which increases NADPH production and elevates intracellular glutathione levels [16]. Additionally, SIRT5 mitigates oxidative stress by suppressing hydrogen peroxide (H₂O₂) production through inhibition of the peroxisomal enzyme Acyl-CoA oxidase 1 [17]. SIRT5 has also been shown to act synergistically with Sirtuin 3 in regulating ROS homeostasis and mitochondrial stability under oxidative conditions [18]. Although the antioxidative functions of SIRT5 have been documented in various cell types, whether it alleviates oxidative stress in bovine mammary glands by regulating mitochondrial fusion and fission remains to be elucidated.

Although existing studies suggest that SIRT5 exerts antioxidative effects, in the context of *S. aureus* infection, how *S. aureus* triggers mitochondrial stress and through which pathway SIRT5 mediates and drives the ensuing oxidative stress and cellular senescence remain inadequately defined. Given that dysregulated mitochondrial dynamics can amplify ROS and create a vicious cycle of persistent damage, and that SIRT5 is mitochondria-localized with desuccinylase activity, this study aims to elucidate the mechanistic role of SIRT5 in regulating mitochondrial fusion and fission in bovine mammary epithelial cells, and to determine its function in alleviating mammary oxidative stress and cellular senescence. These findings are expected to provide new molecular targets and antioxidative intervention strategies for the prevention and control of bovine mastitis.

## Results

### 1. *S. aureus* infection induces mitochondrial ROS accumulation and oxidative stress

**Fig 1:**
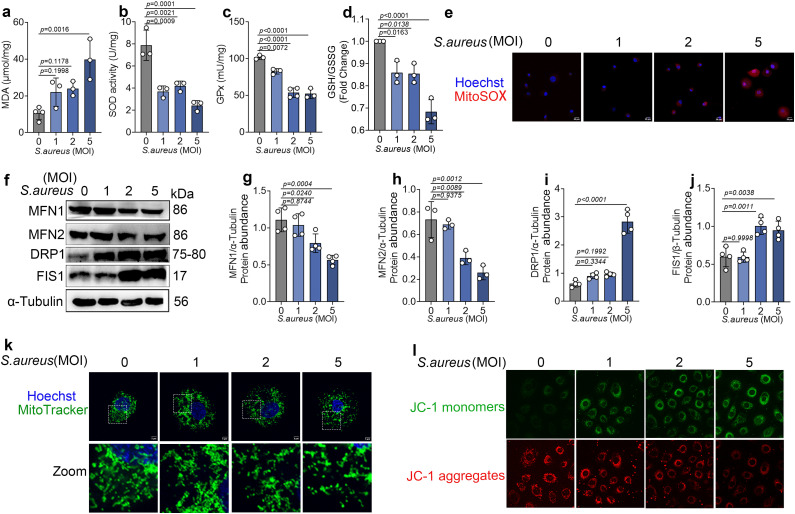
*S.* *aureus* infection induces mitochondrial ROS accumulation and oxidative stress. (a-d) The levels of MDA, SOD, GPx and GSH/GSSG in bovine mammary epithelial cells (n = 3). (e) Mitochondrial superoxide was detected using MitoSOX Red, and nuclei were counterstained with Hoechst. Fluorescence images were acquired using a laser scanning confocal microscope. (n = 3). Scale bar = 20 μm. (f-j) expression levels of mitochondria fusion and fission-related molecules MFN1, MFN2, DRP1, and Fis1 in total cell protein (n = 3). (k) mitochondria were labeled with MitoTracker Green, and nuclei were stained with Hoechst. A laser confocal microscope was used for observation (n = 3). Scale bar = 5 μm. (l) bovine mammary epithelial cells were stained using a JC-1 commercial kit. Data are presented as mean ± SD. Statistical significance among different concentrations was assessed by ordinary one-way ANOVA followed by Tukey’s multiple-comparisons test. *p* values are indicated in the figures.

In this study, bovine mammary epithelial cells were infected with *S. aureus* at different multiplicities of infection (MOI) for 6 h, and oxidative stress–related parameters, including Malondialdehyde (MDA), superoxide dismutase (SOD), glutathione peroxidase (GPx), and the reduced glutathione (GSH) to oxidized glutathione (GSSG) ratio, were measured using commercial assay kits. As MOI increased, *S. aureus* infection caused a significant elevation in MDA levels, whereas SOD, GPx, and the GSH/GSSG ratio decreased in a clear dose-dependent manner (Fig 1a–1d). In addition, the fluorescence intensity of the mitochondria-specific superoxide probe MitoSOX was markedly increased, further indicating that *S. aureus* infection induces mitochondrial ROS accumulation (Fig 1e).

To assess mitochondrial function, we first examined the expression of key mitochondrial fusion and fission proteins. The fusion proteins mitofusin 1 (MFN1) and mitofusin 2 (MFN2) were significantly downregulated, whereas the fission proteins dynamin-related protein 1 (DRP1) and fission 1 (FIS1) were markedly upregulated following infection (Fig 1f–1j). We then used MitoTracker Green to label mitochondria and visualize mitochondrial morphology. In control cells, mitochondria appeared elongated and interconnected, forming a reticular network. By contrast, at MOI = 5, mitochondria became fragmented and appeared as punctate structures (Fig 1k). To further evaluate mitochondrial functional integrity, we assessed mitochondrial membrane potential using the JC-1 assay. In control cells, JC-1 fluorescence was dominated by aggregates with strong signal and relatively weak monomer fluorescence, indicative of normal mitochondrial membrane potential. At MOI = 5, monomer fluorescence was markedly increased, whereas aggregate fluorescence was clearly reduced, consistent with loss of mitochondrial membrane potential (Fig 1l).

Taken together, these findings demonstrate that *S. aureus* infection induces pronounced oxidative stress in bovine mammary epithelial cells and is accompanied by substantial structural and functional mitochondrial damage.

### 2. *S. aureus*–induced mitochondrial stress is predominantly driven by bacterial toxins and inflammatory injury

**Fig 2:**
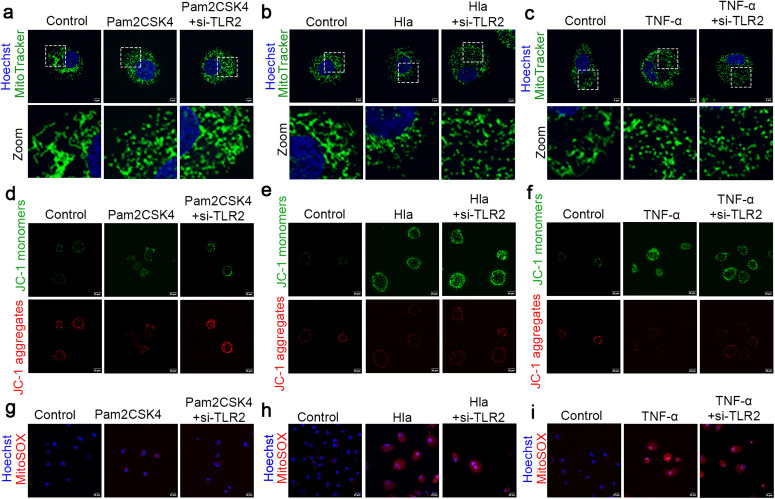
*S.* *aureus*–induced mitochondrial stress is predominantly driven by bacterial toxins and inflammatory injury. (a-c) mitochondria were labeled with MitoTracker Green, and nuclei were stained with Hoechst. A laser confocal microscope was used for observation (n = 3). Scale bar = 5 μm. (d-f) bovine mammary epithelial cells were stained using a JC-1 commercial kit. (g-j) Mitochondrial superoxide was detected using MitoSOX Red, and nuclei were counterstained with Hoechst. Fluorescence images were acquired using a laser scanning confocal microscope. (n = 3). Scale bar = 20 μm.

To explore the mechanisms by which *S. aureus* induces mitochondrial stress, we selected several representative stimuli, including the Toll-like receptor 2 (TLR2) agonist Pam3CSK4, the canonical *S. aureus* toxin α-hemolysin (Hla), the proinflammatory cytokine Tumor Necrosis Factor-α (TNF-α), and the bacterial metabolic product lactate. Because both toxins and inflammatory responses may act through pattern-recognition receptors, si-TLR2 was included as a parallel intervention in the Hla and TNF-α-treated groups. Examination of mitochondrial morphology showed that Pam3CSK4 treatment caused partial mitochondrial fragmentation into punctate structures, which was partially reversed by si-TLR2 (Fig 2a). By contrast, both Hla and TNF-α induced a pronounced fragmented mitochondrial phenotype, and si-TLR2 had no obvious rescuing effect (Fig 2b and 2c). Treatment with sodium L-lactate alone did not alter mitochondrial morphology (S2a Fig). Mitochondrial membrane potential was then assessed using the JC-1 probe by monitoring the monomer/aggregate fluorescence ratio. Pam3CSK4 induced only a mild decrease in membrane potential, which was reversed by si-TLR2 (Fig 2d), whereas Hla and TNF-α caused a marked loss of mitochondrial membrane potential that could not be rescued by si-TLR2 (Fig 2e and 2f). Lactate treatment had no detectable effect on membrane potential (S2b Fig). Finally, mitochondrial superoxide levels were evaluated using MitoSOX. Neither Pam3CSK4 alone nor Pam3CSK4 plus si-TLR2 produced a clear increase in MitoSOX fluorescence (Fig 2g). In contrast, both Hla and TNF-α markedly elevated MitoSOX fluorescence intensity, and this effect was not attenuated by si-TLR2 (Fig 2h and 2i). Lactate likewise failed to increase MitoSOX signal (S2c Fig).

Taken together, these findings indicate that bacterial toxins and inflammatory mediators are the major drivers of *S. aureus*–induced mitochondrial stress, whereas TLR2-mediated pattern recognition, although contributing to the response, is not the principal determinant.

### 3. The *S. aureus* toxin Hla elicits an early inflammatory response followed by secondary mitochondrial stress

**Fig 3:**
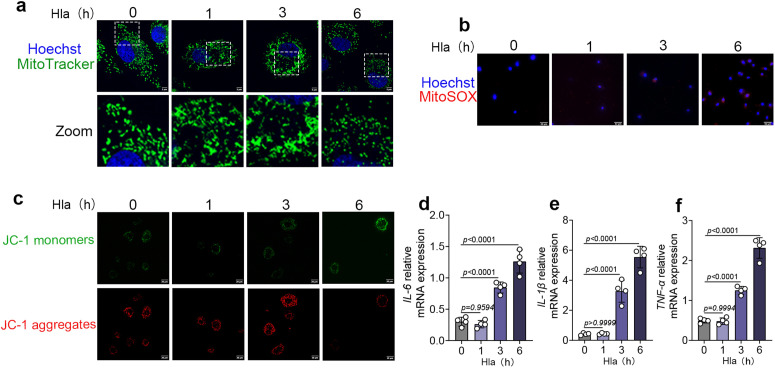
The *S. aureus* toxin Hla elicits an early inflammatory response followed by secondary mitochondrial stress. (a) mitochondria were labeled with MitoTracker Green, and nuclei were stained with Hoechst. A laser confocal microscope was used for observation (n = 3). Scale bar = 5 μm. (b) Mitochondrial superoxide was detected using MitoSOX Red, and nuclei were counterstained with Hoechst. Fluorescence images were acquired using a laser scanning confocal microscope. (n = 3). Scale bar = 50 μm. (c) bovine mammary epithelial cells were stained using a JC-1 commercial kit. (d-f) *IL-6, IL-1β* and *TNF-α* mRNA expression level. Data are presented as mean ± SD. Statistical significance among different concentrations was assessed by ordinary one-way ANOVA followed by Tukey’s multiple-comparisons test. *p* values are indicated in the figures.

To further clarify the underlying mechanism, cells were stimulated with Hla and mitochondrial stress and inflammatory responses were assessed at different time points. At 0, 1, and 3 h after stimulation, mitochondrial morphology, MitoSOX fluorescence, and mitochondrial membrane potential showed no evident changes; however, by 6 h a typical mitochondrial stress phenotype emerged, characterized by excessive mitochondrial fragmentation, increased MitoSOX signal, and loss of mitochondrial membrane potential (Fig 3a–3c). In contrast, mRNA levels of the inflammatory cytokines *IL-6*, *IL-1β*, and *TNF-α* were already significantly elevated at 3 h and continued to increase at 6 h (Fig 3d). These findings indicate that, under the action of the *S. aureus* toxin, activation of the inflammatory response precedes the onset of mitochondrial stress, that is, the toxin initially induces inflammatory injury, which subsequently triggers mitochondrial stress.

### 4. Mitochondrial stress exacerbates *S. aureus* infection and promotes cellular senescence responses

**Fig 4:**
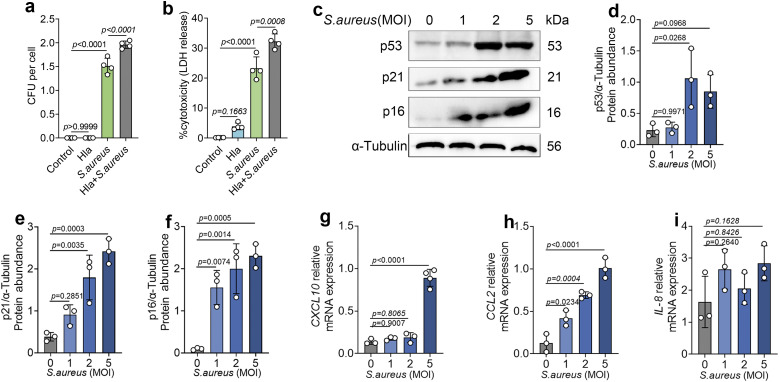
Mitochondrial stress exacerbates *S. aureus* infection and promotes cellular senescence responses. (a) Intracellular bacterial load expressed as CFU per cell (n = 4). (b) Cell injury determined by LDH release and presented as percentage cytotoxicity (n = 4). (c-f) Expression levels of senescence-associated molecules p53, p21, and p16 in total cell protein (n = 3). (g-i) mRNA expression levels of senescence-associated secretory factors *CXCL10*, *CCL2*, and *IL-8* in mammary epithelial cells (n = 3). Data are presented as mean ± SD. Statistical significance among different concentrations was assessed by ordinary one-way ANOVA followed by Tukey’s multiple-comparisons test. *p* values are indicated in the figures.

In the experiments described above, we established that *S. aureus* induces mitochondrial stress and delineated its upstream regulatory mechanisms. We next investigated whether this mitochondrial stress response exerts a protective effect on cell fate or merely represents a pathological insult. To this end, cells were pretreated with Hla to induce mitochondrial stress prior to *S. aureus* infection, and intracellular bacterial burden and cell status were subsequently assessed. Compared with *S. aureus* infection alone, cells subjected to mitochondrial stress exhibited a significantly higher intracellular bacterial load following infection (Fig 4a). Using Lactate Dehydrogenase (LDH) release as an indicator of cell damage and death, we found that LDH release was markedly increased in mitochondrially stressed cells after infection, indicating aggravated cellular injury (Fig 4b). These results suggest that *S. aureus*–induced mitochondrial stress is not a protective adaptive response, but rather a pathological process that amplifies cellular damage and compromises the antibacterial and self-protective capacity of bovine mammary epithelial cells during infection.

Mitochondrial dysfunction has been reported in multiple studies to directly contribute to cellular senescence [19,20]. Therefore, we evaluated the senescence status of bovine mammary epithelial cells following *S. aureus* infection. The results showed significant upregulation of senescence markers p53, p21, and p16 after *S. aureus* stimulation (Fig 4c–4f). In addition, SASP factors, including *CXCL10* and *CCL2*, were significantly increased, whereas *IL-8* showed only a modest upward trend without reaching statistical significance (Fig 4g–4i). These findings indicate that *S. aureus* infection drives senescence in bovine mammary epithelial cells.

### 5. *S. aureus* infection promotes SIRT5 degradation by enhancing its interaction with UBC

**Fig 5:**
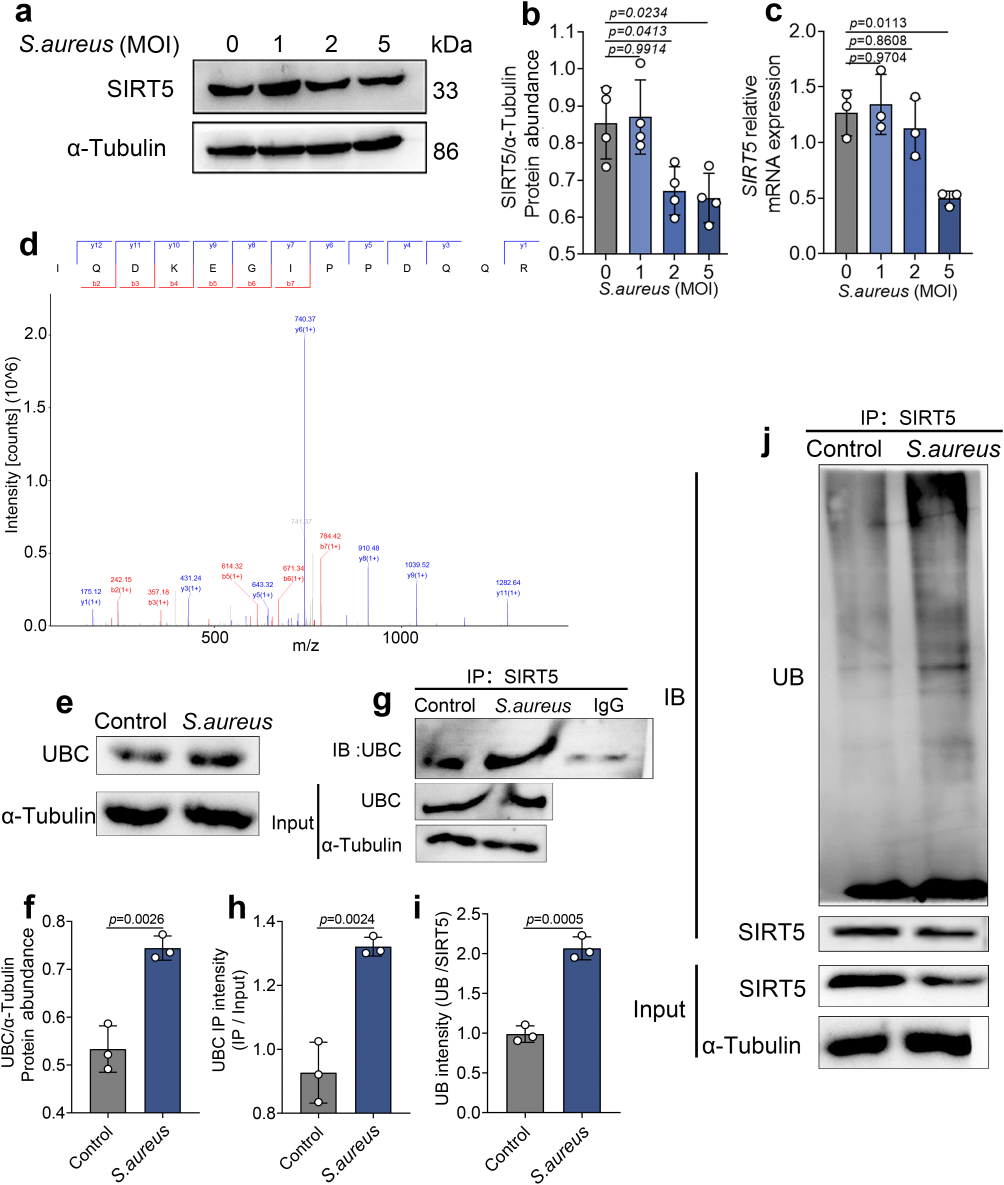
*S.* *aureus* infection promotes SIRT5 degradation by enhancing its interaction with UBC. (a–b) protein abundance levels of SIRT5 (n = 4). (c) mRNA expression level of *SIRT5* (n = 3). (d) Mass spectrometry identification spectrum of UBC; (e, f) protein abundance level of UBC. (g, h) Co-IP assay verifying the interaction between SIRT5 and UBC. (i. j) Ubiquitination level of SIRT5. Data are presented as mean ± SD. Statistical significance for the concentration–response experiments in panels b–c was assessed by ordinary one-way ANOVA followed by Tukey’s multiple-comparisons test. Statistical significance for the two-group comparisons in panels f–i was assessed by a two-tailed unpaired Student’s t-test. *p* values are indicated in the figure.

We next examined the protein and mRNA expression levels of SIRT5 in this process and found that oxidative stress induced by *S. aureus* infection significantly downregulated SIRT5 expression (Fig 5a–5c). Interestingly, SIRT5 protein levels declined noticeably at an MOI of 2, whereas its mRNA levels decreased only at an MOI of 5 (Fig 5c). This observation suggests that the reduction in SIRT5 protein levels may not be solely due to transcriptional regulation but may involve other regulatory mechanisms. To further explore the potential mechanism, we immunoprecipitated SIRT5 from *S. aureus*–infected bovine mammary epithelial cells and identified its interacting proteins by mass spectrometry. The results revealed an interaction between SIRT5 and Ubiquitin C (UBC) (Fig 5d). We then performed a follow‑up Co-immunoprecipitation (Co‑IP) assay to validate this interaction and found that the binding between SIRT5 and UBC was significantly enhanced under infection (Fig 5e and 5f). Meanwhile, UBC also exhibited an upward trend at the total protein level (Fig 5g and 5h). Given that UBC plays a key role in protein ubiquitination, we further examined the ubiquitination level of SIRT5 under infection at an MOI of 2. The results demonstrated that SIRT5 ubiquitination was significantly increased (Fig 5i and 5j). Together, these findings suggest that *S. aureus* infection may enhance the interaction between UBC and SIRT5, promoting SIRT5 ubiquitination and degradation, thereby leading to reduced SIRT5 protein levels.

### 6. SIRT5 regulates mitochondrial fission by modulating the succinylation of DRP1

To investigate the potential mechanism underlying *S. aureus*–induced excessive mitochondrial fission, we focused on the key function of SIRT5—its ability to desuccinylate target proteins. We generated a SIRT5 overexpression plasmid (S3 Fig.) and transfected it into bovine mammary epithelial cells. Cells were then infected with *S. aureus* at MOI = 5 for 6 h to establish the infection model.

**Fig 6:**
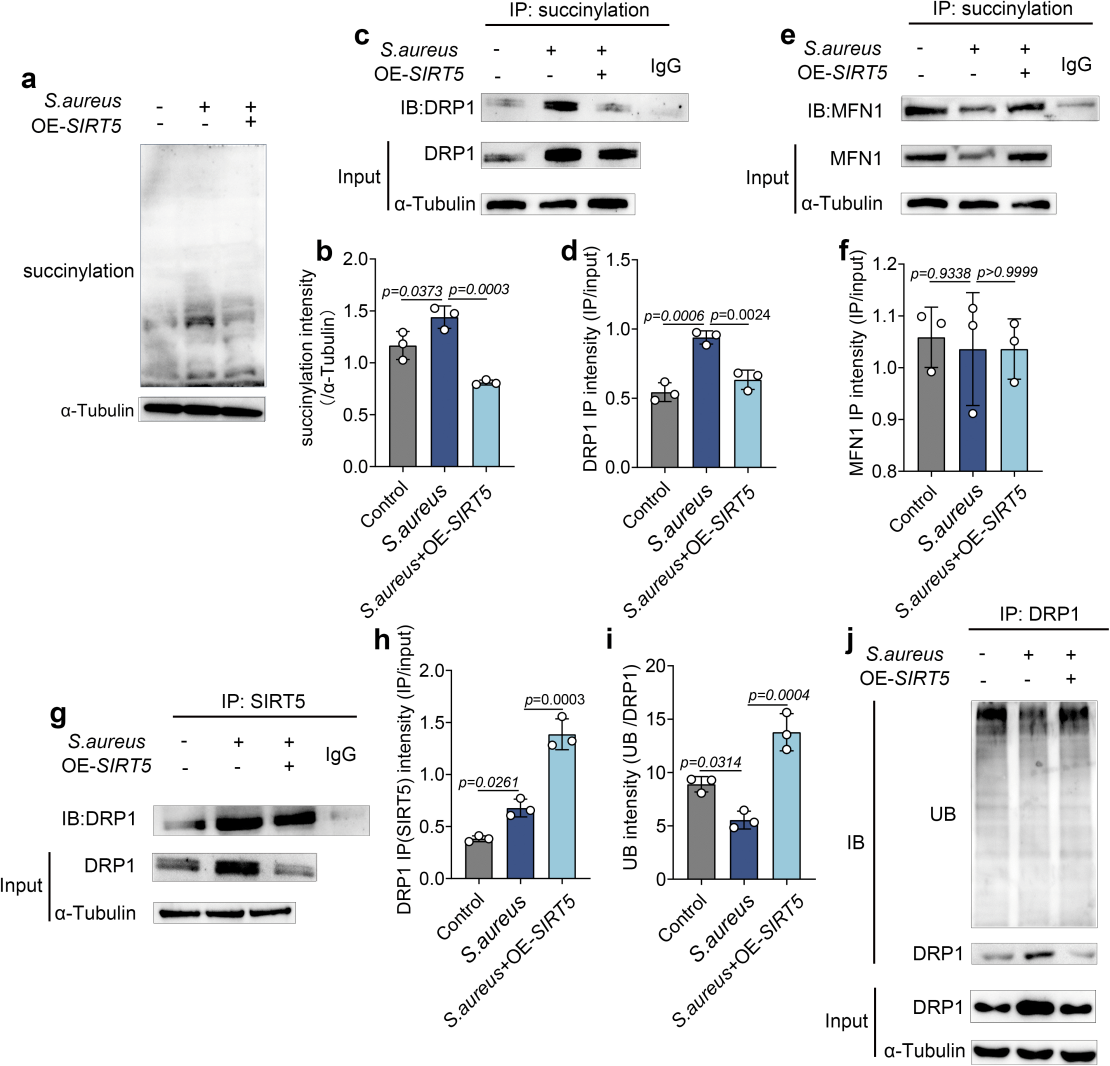
SIRT5 regulates mitochondrial fission by modulating the succinylation of DRP1. (a,b) Detection of total succinylation levels in mammary epithelial cells. (c, d) immunoprecipitation assay to detect the succinylation level of DRP1. (e, f) immunoprecipitation assay to detect the succinylation level of MFN1. (g, h) Co-IP assay to detect the interaction between SIRT5 and DRP1. (i, j) immunoprecipitation assay to detect the ubiquitination level of DRP1. Data are presented as mean ± SD. Statistical significance was assessed by ordinary one-way ANOVA followed by Tukey’s multiple-comparisons test. *p* values are indicated in the figure.

We first measured the global succinylation level and found that it was significantly increased after *S. aureus* infection, whereas overexpression of SIRT5 markedly reduced it (Fig 6a and 6b). Since excessive mitochondrial fission could result either from enhanced fission or from impaired fusion, we next examined the succinylation levels of the key fission protein DRP1 and the key fusion protein MFN1 (Fig 6c-6f). The results showed that DRP1 succinylation was significantly elevated after *S. aureus* infection and significantly decreased upon SIRT5 overexpression; in contrast, MFN1 succinylation was unaffected by either *S. aureus* infection or SIRT5 overexpression. Based on these findings, we focused subsequent investigations on DRP1. To further determine whether SIRT5 exerts its function through DRP1, we performed Co‑IP assays to assess their interaction. We found that SIRT5 interacted with DRP1, and this interaction persisted under infection conditions (Fig 6g and 6h). In addition, to explore the regulatory mechanism of DRP1 expression changes, we examined its ubiquitination level. We found that DRP1 ubiquitination was reduced following *S. aureus* infection, whereas overexpression of SIRT5 reversed this effect and significantly increased DRP1 ubiquitination (Fig 6i and 6j). Collectively, these findings suggest that *S. aureus* infection downregulates SIRT5 expression, leading to enhanced DRP1 succinylation and inhibition of its ubiquitin‑mediated degradation, resulting in DRP1 accumulation on the mitochondrial outer membrane and ultimately driving excessive mitochondrial fission.

### 7. SIRT5 attenuates *S. aureus*–induced mitochondrial stress in bovine mammary epithelial cells

**Fig 7:**
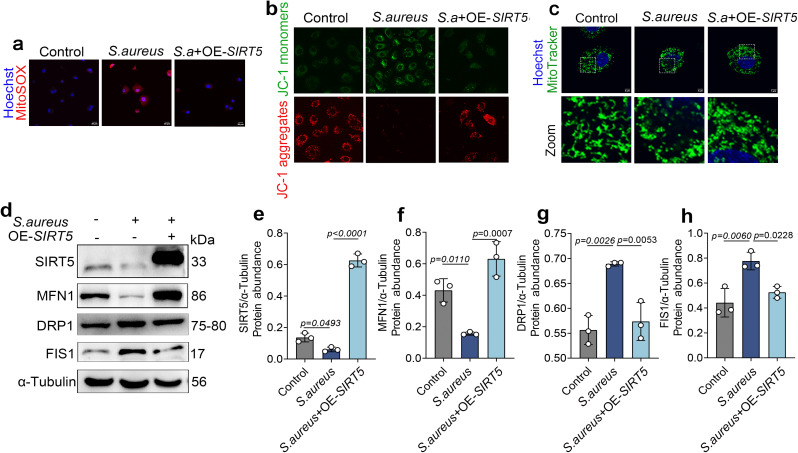
SIRT5 attenuates *S. aureus*–induced mitochondrial stress in bovine mammary epithelial cells. (a) Mitochondrial superoxide was detected using MitoSOX Red, and nuclei were counterstained with Hoechst. Fluorescence images were acquired using a laser scanning confocal microscope. (n = 3). Scale bar = 20 μm. (b) bovine mammary epithelial cells were stained using a JC-1 commercial kit; (c) mitochondria were labeled with MitoTracker Green, and nuclei were stained with Hoechst. A laser confocal microscope was used for observation (n = 3). Scale bar = 5 μm. (d-h) protein abundance levels of MFN1, DRP1, and FIS1. Data are presented as mean ± SD. Statistical significance was assessed by ordinary one-way ANOVA followed by Tukey’s multiple-comparisons test. *p* values are indicated in the figure.

These results indicate that SIRT5 regulates mitochondrial fission by modulating the succinylation status of DRP1. We next performed functional studies to evaluate the impact of SIRT5 on mitochondrial stress. Mitochondrial oxidative stress was first assessed using MitoSOX. SIRT5 overexpression markedly attenuated the *S. aureus*–induced increase in MitoSOX fluorescence (Fig 7a). Assessment of mitochondrial membrane potential by JC-1 staining showed that *S. aureus* infection increased JC-1 monomer fluorescence and decreased aggregate fluorescence, indicating loss of mitochondrial membrane potential, whereas SIRT5 overexpression substantially alleviated this damage (Fig 7b). Consistently, MitoTracker-based analysis of mitochondrial morphology demonstrated that SIRT5 overexpression clearly mitigated the excessive mitochondrial fragmentation induced by *S. aureus* (Fig 7c).

To further substantiate these observations, we examined the expression of key mitochondrial fusion and fission proteins by western blot. SIRT5 overexpression reversed the *S. aureus*–induced downregulation of the fusion protein MFN1 and the upregulation of the fission proteins DRP1 and FIS1 (Fig 7d–7h). Taken together, these findings indicate that SIRT5 alleviates mitochondrial stress in bovine mammary epithelial cells during *S. aureus* infection.

### 8. SIRT5 attenuates *S. aureus*–induced oxidative stress and senescence in bovine mammary epithelial cells

**Fig 8:**
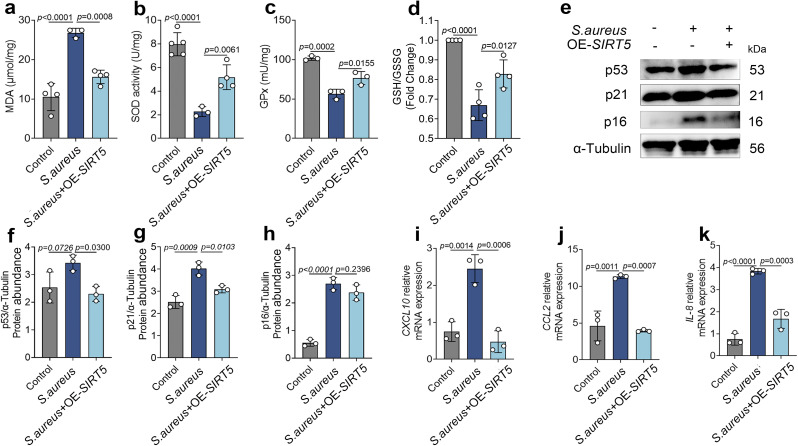
SIRT5 attenuates *S. aureus*–induced oxidative stress and senescence in bovine mammary epithelial cells. (a-d) levels of MDA, SOD, GPx and GSH/GSSG in bovine mammary epithelial cells (n = 4). (e-h) Expression levels of senescence-associated molecules p53, p21, and p16 in total cell protein (n = 3). (i-k) mRNA expression levels of senescence-associated secretory factors *CXCL10*, *CCL2*, and *IL-8* in mammary epithelial cells (n = 3). Data are presented as mean ± SD. Statistical significance was assessed by ordinary one-way ANOVA followed by Tukey’s multiple-comparisons test. *p* values are indicated in the figure.

Subsequently, oxidative stress–related indicators—including MDA, SOD, GPx and GSH/GSSG—were measured using commercial assay kits to evaluate the effect of SIRT5 on cellular redox status under infection conditions. The results showed that compared with the oxidative stress model group, SIRT5 overexpression significantly reduced MDA levels while significantly increasing SOD, GPx and GSH/GSSG levels in bovine mammary epithelial cells (Fig 8a-8d), suggesting a protective role of SIRT5 against oxidative stress. We further analyzed the expression levels of senescence‑related marker proteins. Western blot results showed that compared with the infection group, SIRT5 overexpression significantly reduced the protein levels of p53, p16, and p21 (Fig 8e-8h). In addition, the marked upregulation of SASP factors *CXCL10*, *CCL2*, and *IL‑8* observed in the infection group was also reversed by SIRT5 overexpression (Fig 8i-8k). These findings indicate that SIRT5 effectively delays senescence of bovine mammary epithelial cells under *S. aureus*–induced stress conditions.

### 9. Excessive mitochondrial fission attenuates the ability of SIRT5 to alleviate oxidative stress and senescence

**Fig 9:**
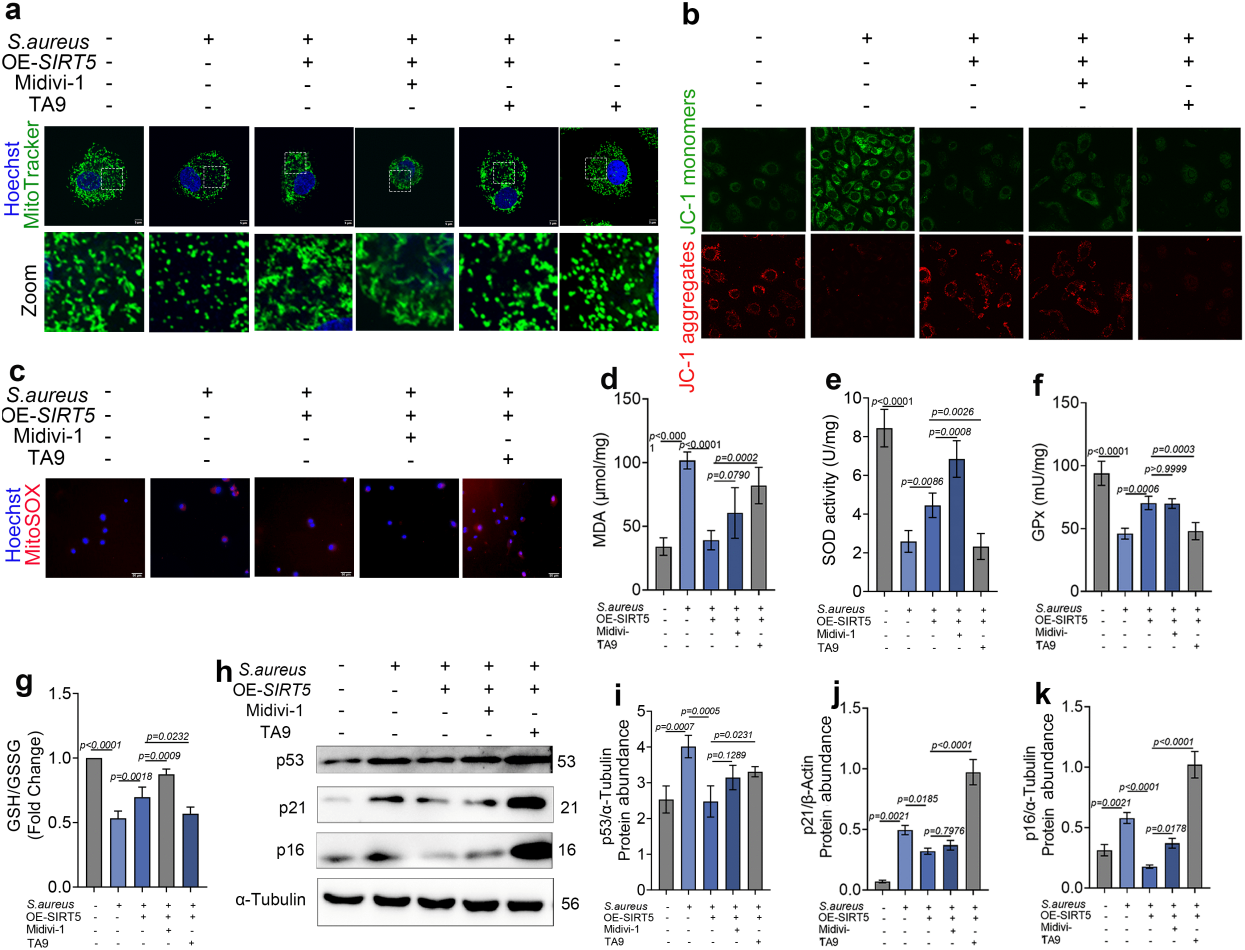
Excessive mitochondrial fission attenuates the ability of SIRT5 to alleviate oxidative stress and senescence. (a) mitochondria were labeled with MitoTracker Green, and nuclei were stained with Hoechst. A laser confocal microscope was used for observation (n = 3). Scale bar = 5 μm. (b) bovine mammary epithelial cells were stained using a JC-1 commercial kit. (c) Mitochondrial superoxide was detected using MitoSOX Red, and nuclei were counterstained with Hoechst. Fluorescence images were acquired using a laser scanning confocal microscope. (n = 3). Scale bar = 50 μm. (d-g) levels of MDA, SOD, GPx and GSH/GSSG in bovine mammary epithelial cells (n = 4). (h-k) Expression levels of senescence-associated molecules p53, p21, and p16 in total cell protein (n = 3). Data are presented as mean ± SD. Statistical significance was assessed by ordinary one-way ANOVA followed by Tukey’s multiple-comparisons test. *p* values are indicated in the figure.

To further validate the role of the UBC/SIRT5/DRP1 axis in *S. aureus*–induced mitochondrial stress, we pharmacologically manipulated mitochondrial fission. Mitochondrial morphology analysis confirmed that Mdivi-1 inhibited mitochondrial fission and induced a hyperfused mitochondrial network, whereas Tyrphostin A9 (TA9) treatment caused pronounced mitochondrial fragmentation. *S. aureus* infection also led to mitochondrial fragmentation but, in contrast to TA9, was accompanied by a reduction in overall MitoTracker fluorescence, suggesting a decrease in mitochondrial mass and indicating that the patterns of damage induced by TA9 and *S. aureus* are not identical (Fig 9a).

On this basis, Mdivi-1 or TA9 was applied in the context of SIRT5 overexpression, followed by *S. aureus* infection. JC-1 staining showed that co-treatment with Mdivi-1 produced little change compared with the SIRT5 + *S. aureus* group, whereas TA9-induced excessive fission caused a renewed decline in mitochondrial membrane potential, markedly weakening the protective effect of SIRT5 (Fig 9b). Consistently, MitoSOX analysis revealed that Mdivi-1 did not compromise this protective effect, whereas TA9 restored the increase in MitoSOX fluorescence (Fig 9c). Moreover, relative to SIRT5 overexpression alone, addition of Mdivi-1 did not significantly alter oxidative stress indices, while TA9 significantly increased MDA levels and reduced SOD and GPx activities and the GSH/GSSG ratio (Fig 9d–9g), indicating that excessive mitochondrial fission diminishes the ability of SIRT5 to mitigate oxidative stress.

Similarly, compared with SIRT5 overexpression alone, Mdivi-1 had no significant effect on the expression of the senescence-associated proteins p53, p16, and p21, whereas TA9 markedly upregulated these proteins (Fig 9h-9k), suggesting that excessive mitochondrial fission also impairs the ability of SIRT5 to attenuate bovine mammary epithelial cell senescence. Overall, the capacity of SIRT5 to improve mitochondrial function, reduce oxidative stress, and alleviate cellular senescence depends on a relatively moderate level of DRP1-mediated fission. When mitochondrial fission is excessively activated, the cytoprotective effect of SIRT5 is substantially weakened, consistent with our proposed model in which the UBC/SIRT5/DRP1 axis regulates mitochondrial dynamics.

### 10. *S. aureus* infection induces severe oxidative stress and cellular senescence, accompanied by downregulation of SIRT5 expression

**Fig 10:**
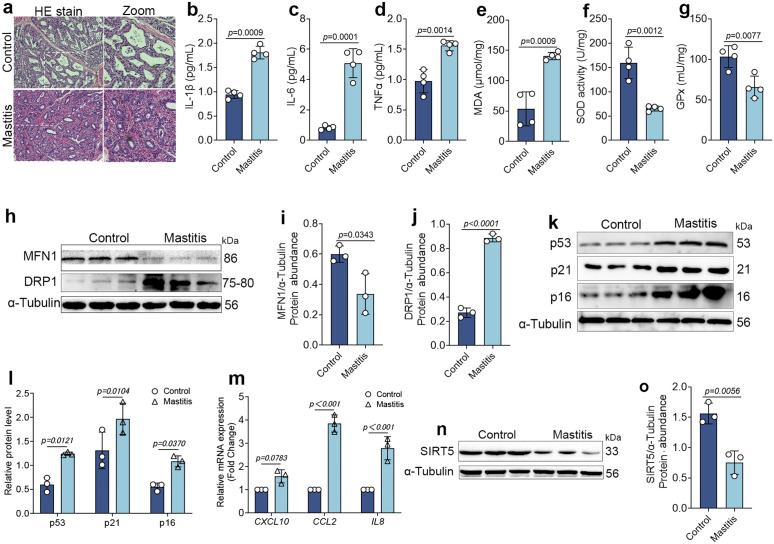
*S. aureus* infection induces severe oxidative stress and cellular senescence, accompanied by downregulation of SIRT5 expression. (a) HE staining was used to assess the level of mammary gland injury in bovine mammary tissue (n = 3), scale bar: 200 μm. (b-d) ELISA was used to detect the contents of IL-1β, IL-6, and TNF-α in bovine mammary tissue (n = 4). (e-g) contents of MDA, SOD, and GPx in bovine mammary tissue (n = 4). (h-j) protein abundance levels of mitochondria fusion and fission-related molecules MFN1 and DRP1 in bovine mammary tissue (n = 3). (k, l) protein abundance levels of senescence-associated molecules p53, p21, and p16 in bovine mammary tissue (n = 3). (m) mRNA expression levels of senescence-associated secretory factors *CXCL10*, *CCL2*, and *IL-8* in bovine mammary tissue (Fold Change) (n = 3). (n, o) protein abundance levels of SIRT5 in bovine mammary tissue (n = 3). Data are presented as mean ± SD. Statistical significance was assessed by a two-tailed unpaired Student’s t-test. *p* values are indicated in the figure.

Finally, to determine whether the inflammation, mitochondrial stress, and senescence observed in vitro were recapitulated in vivo, we analyzed mammary tissues from healthy cows and cows with Staphylococcus aureus–associated mastitis. Hematoxylin and eosin staining revealed pronounced mammary alveolar atrophy, thickened alveolar walls, and massive infiltration of inflammatory cells in mastitic mammary tissues (Fig 10a). Consistently, ELISA results showed that the levels of proinflammatory cytokines IL‑1β, IL‑6, and TNF‑α were significantly elevated in the mastitis group compared to healthy controls (Fig 10b–10d), indicating marked inflammation in the mammary gland.

Next, we quantified oxidative stress markers in mammary tissues using commercial assay kits. MDA levels were significantly increased, while the antioxidant enzymes SOD and GPx were significantly decreased in mastitic cows (Fig 10e–10g), suggesting that mammary tissues were in a state of oxidative stress. Given the critical role of mitochondrial integrity in maintaining redox balance, we examined the expression of the key mitochondrial dynamics proteins MFN1 and DRP1. We found that MFN1 was markedly downregulated, whereas DRP1 was significantly upregulated in mastitic tissues (Fig 10h–10j).

Senescence of mammary tissue is closely related to recovery and prognosis of bovine mastitis. We observed significant upregulation of senescence markers p53, p21, and p16 in mastitic mammary tissues (Fig 10k, 10l). Moreover, the expression of SASP factors *CXCL10*, *CCL2*, and *IL‑8* was also markedly increased (Fig 10m), indicating the onset of cellular senescence during mastitis. Finally, we assessed mitochondrial SIRT5 levels and found a pronounced downregulation of SIRT5 in mastitic mammary tissues (Fig 10n and 10o).

Taken together, these in vivo findings closely mirror the *S. aureus*–induced inflammation, mitochondrial dysfunction, and cellular senescence observed in our in vitro model. They further suggest that SIRT5 downregulation, imbalanced mitochondrial dynamics, and cellular senescence are tightly associated with the onset and progression of *S. aureus*–related bovine mastitis.

## Discussion

Bovine mastitis is one of the most prevalent and economically devastating diseases in dairy farms, with *S. aureus* infection being a major causative agent [21]. This study focused on the regulatory role of SIRT5 in *S. aureus*–induced oxidative stress and senescence in bovine mammary epithelial cells and revealed a novel mechanism by which the UBC/SIRT5/DRP1 axis regulates mitochondrial dynamics. These findings provide important theoretical insights for the prevention and treatment of mastitis.

Our results demonstrated that *S. aureus* infection induced significant oxidative stress in bovine mammary tissues and epithelial cells, as evidenced by increased MDA levels, decreased activities of SOD and GPx, and a reduced GSH/GSSG ratio. In parallel, mitochondrial stress phenotypes were observed, including fragmented mitochondrial morphology, downregulation of the fusion protein MFN1, upregulation of the fission protein DRP1, increased mitochondrial superoxide, and a significant reduction in mitochondrial membrane potential. These observations are consistent with previous reports, indicating that *S. aureus* disrupts cellular redox homeostasis through multiple pathways, induces excessive mitochondrial fission, and triggers mitochondrial stress responses. As the central organelles of cellular energy metabolism, mitochondria, once structurally and functionally perturbed, further exacerbate oxidative stress, thereby establishing a vicious cycle of “mitochondrial damage–oxidative stress.” In conjunction with the mechanistic experiments in this study, we further identified *S. aureus*–derived toxins and the resultant inflammatory response as the principal drivers of mitochondrial stress among several potential contributing factors, whereas Pam-mediated TLR2 activation and lactate metabolism per se exerted only limited effects on mitochondrial injury. Moreover, we demonstrated that Hla rapidly triggers a robust increase in proinflammatory cytokines (including TNF-α). In contrast, mitochondrial stress became evident at later time points, as indicated by excessive mitochondrial fragmentation, loss of mitochondrial membrane potential, and increased mitochondrial superoxide production. Given that TNF-α and related cytokines were induced as early as 3 h, whereas mitochondrial phenotypes were not apparent until 6 h, it is plausible that Hla-driven mitochondrial stress is mediated indirectly, at least in part, through autocrine/paracrine inflammatory signaling rather than being solely attributable to direct toxin activity. In this niche, therefore, Hla may function not only as a cytolytic toxin but also as an inflammation-amplifying factor that creates a permissive, hyperinflammatory microenvironment, which in turn exacerbates mitochondrial dysfunction and oxidative injury in mammary epithelial cells. Our time-course data support this interpretation and underscore a cooperative mode of action in which toxin-triggered inflammation precedes and potentiates mitochondrial stress. Hla is one of the major virulence factors of *S. aureus*, and previous studies have shown that it forms pores in the plasma membrane, leading to potassium efflux and elevated reactive oxygen species, thereby activating the MAPK–NLRP3 axis and triggering inflammatory responses [22,23]. Our findings are in line with these reports and further suggest that Hla–mediated amplification of inflammation acts as a critical bridge linking *S. aureus* infection to mitochondrial stress.

In this process, we noted a significant downregulation of SIRT5 expression. As a mitochondrial-localized desuccinylase, SIRT5 has been shown to be closely associated with the regulation of oxidative stress responses and mitochondrial function [18,24–26]. Therefore, SIRT5 may act as a molecular bridge between *S. aureus*–induced mitochondrial dynamics abnormalities and cellular oxidative stress, serving as a critical mediator of mitochondrial structural alterations that lead to oxidative stress and senescence. Based on this, after establishing that *S. aureus* infection induces cellular damage and mitochondrial dysfunction, we further focused on the key regulatory factor SIRT5 to elucidate its potential mechanism in this process.

We found that *S. aureus* infection significantly reduced SIRT5 protein levels, whereas its mRNA levels only decreased at higher infection intensities, suggesting that its downregulation may involve protein homeostasis regulation. Additional evidence indicated that under low‑dose infection, UBC levels were elevated and its interaction with SIRT5 was enhanced, accompanied by increased SIRT5 ubiquitination, suggesting that UBC promotes SIRT5 degradation during this process. Previous studies have shown that the SCF^Cyclin F complex also regulates SIRT5 stability via ubiquitination [27], and UBC, as a “ubiquitin reservoir” donor in oxidative stress environments, helps clear damaged proteins and maintain homeostasis [28,29]. Our findings not only support the notion that Sirtuins family proteins are commonly constrained by ubiquitination during oxidative stress [30,31] but also suggest that the UBC–SIRT5 interaction may represent a potential therapeutic target to protect mammary epithelial cells from oxidative damage.

Our results demonstrated that SIRT5 interacts with DRP1 and modulates its succinylation level. Further investigations revealed that *S. aureus*–induced downregulation of SIRT5 not only increased DRP1 succinylation but also reduced its ubiquitin‑mediated degradation. These findings suggest that *S. aureus* infection promotes excessive mitochondrial fission by downregulating SIRT5, thereby inhibiting DRP1 degradation. Previous studies have shown that succinylation can influence protein ubiquitination and stability [32,33]. For example, in a sepsis‑induced cardiac dysfunction model, succinylation at lysine 352 of SERCA2a facilitated the formation of K48‑linked ubiquitin chains and proteasomal degradation [34]. In contrast, another study reported that succinylation at lysine 222 of Lactate Dehydrogenase A, despite leaving its overall ubiquitination level unchanged, disrupted its interaction with Sequestosome 1, a ubiquitin‑binding adaptor protein, thereby reducing its lysosomal degradation [35]. In light of our observation that DRP1 succinylation was reduced whereas its ubiquitination increased, we speculate that SIRT5‑regulated succinylation may influence DRP1 conformation or compete for ubiquitin recognition sites, thereby altering its ubiquitination status and regulating the mitochondrial fission process.

To determine whether the above molecular mechanism confers actual protective effects at the cellular level, we further conducted functional validation experiments. The results showed that SIRT5 overexpression significantly alleviated *S. aureus*–induced oxidative stress and mitochondrial dysfunction and delayed the progression of cellular senescence. These functional findings are consistent with the proposed molecular mechanism by which SIRT5 regulates mitochondrial dynamics. The structural and functional integrity of mitochondria is crucial in the processes of oxidative stress and cellular senescence [36,37]. Mitochondrial damage can lead to impaired energy metabolism and sustained ROS accumulation, forming a positive feedback loop that exacerbates cellular and tissue aging [38,39]. In addition, mitochondrial‑targeted antioxidant peptides, such as SS‑31, have been shown to significantly reduce intracellular H₂O₂ levels and improve oxidative phosphorylation efficiency, thereby enhancing cardiac and skeletal muscle function in aged animals [40]. These findings are in line with our proposed mechanism that SIRT5 mitigates infection‑associated oxidative stress and cellular senescence by maintaining mitochondrial homeostasis.

Finally, we performed a reverse validation of this pathway from the downstream side by examining whether SIRT5 could still exert its protective effects under conditions of excessive mitochondrial fission or excessive fusion. Our study demonstrated that excessive mitochondrial fission significantly impaired the ability of SIRT5 to mitigate oxidative stress and cellular senescence, whereas excessive fusion had little effect on SIRT5 function. This suggests that excessive mitochondrial fission interferes with the protective effects of SIRT5. Further analysis revealed that although SIRT5 overexpression could partially suppress oxidative stress–induced mitochondrial fission, once excessive fission was established, SIRT5 might have difficulty precisely localizing or recognizing its substrates, thereby limiting its desuccinylase activity. Previous studies have shown that SIRT5 suppresses oxidative stress by modulating the succinylation of Peroxiredoxin 3 and Apoptosis-Inducing Factor, Mitochondrion-Associated 1 [24,41]. Therefore, we speculate that excessive mitochondrial fission may disrupt SIRT5’s ability to recognize and desuccinylate its key substrates, thereby weakening its protective effects.

The UBC/SIRT5/DRP1 axis identified in this study provides a novel molecular target for the prevention and treatment of bovine mastitis. As a key regulator of mitochondrial dynamics and oxidative stress, activation or stabilization of SIRT5 may represent a new strategy for intervening in mastitis. Developing UBC inhibitors to block the ubiquitin‑mediated degradation of SIRT5 or designing SIRT5 agonists to enhance its desuccinylase activity could be effective approaches for treating mastitis. In addition, targeting the succinylation status of DRP1 also offer a new avenue for improving mitochondrial function and alleviating oxidative stress.

## Conclusion

In summary, this study elucidates a critical mechanism by which SIRT5 regulates mitochondrial fission and oxidative stress during *S. aureus*–induced oxidative stress. We found that, following *S. aureus* infection, bacterial toxins induce an inflammatory response, which activates the ubiquitin-conjugating enzyme UBC, enhances its interaction with SIRT5, and promotes ubiquitin-mediated degradation of SIRT5, leading to a marked reduction in SIRT5 protein levels. Downregulation of SIRT5 results in increased succinylation and decreased ubiquitination of DRP1, causing its accumulation on the mitochondrial membrane and inducing excessive mitochondrial fission. Mitochondrial fragmentation, in turn, impairs the antioxidant function of SIRT5, forming a self‑perpetuating positive feedback loop that ultimately drives mammary epithelial cells into a senescent state. This study provides new insights into the role of SIRT5 in the regulation of infection‑ and senescence‑related processes and offers a theoretical basis and potential targets for developing SIRT5‑based anti‑infection and anti‑senescence strategies.

## Materials and methods

### Ethics statement

The animal research protocol was ethically approved by the Jilin University Committee on Animal Use and Care (Changchun, China; protocol number SY202506008).

### Materials

#### 1. Antibodies.

The following primary antibodies were used for Western blot and co-immunoprecipitation experiments: anti-MFN1 (Proteintech, 13798–1-AP, 1:1000), anti-MFN2 (Proteintech, 12186–1-AP, 1:1000), anti-FIS1 (Proteintech, 10956–1-AP, 1:1000), anti-DRP1 (Proteintech, 12957–1-AP, 1:2000), anti-p53 (Cell Signaling Technology, 9282S, 1:1000), anti-p21 (Santa Cruz Biotechnology, sc-6246, 1:200), anti-p16 (Proteintech, 10883–1-AP, 1:1000), anti-SIRT5 (Proteintech, 15122–1-AP, 1:1000), anti-UBC (Proteintech, 10457–1-AP, 1:1000), anti-ubiquitin (Proteintech, 10201–2-AP, 1:1000), anti-succinyl-lysine (PTM Biolabs, PTM-401, 1:1000), and anti-α-Tubulin (Proteintech, 66031–1-Ig, 1:2000) as the loading control. HRP-conjugated goat anti-rabbit IgG (Proteintech, SA00001–2, 1:10000) and goat anti-mouse IgG (Proteintech, SA00001–1, 1:5000) were used as secondary antibodies.

#### 2. Reagents and Kits.

The following reagents were used in this study: Lidocaine (1366002, Sigma-Aldrich), Lipofectamine 2000 (11668019, Invitrogen Life Technologies), Mdivi-1 (HY-15886, MedChemExpress), TA9 (HY-15511, MedChemExpress), Pam2CSK4 TFA(HY-P1181A,MedChemExpress), Alpha-hemolysin(HY-P71825, MedChemExpress), Recombinant Bovine TNF-α Protein (90645ES10, Yeasen), Sodium lactate (S108838, Aladdin), MDA assay kit (S0131M, Beyotime Biotechnology), SOD assay kit (S0101S, Beyotime Biotechnology), GPx assay kit (S0056, Beyotime Biotechnology), GSH and GSSG assay kit (S0053, Beyotime Biotechnology), LDH assay kit (C0018S, Beyotime Biotechnology), IL-1α assay kit (432615, BioLegend), IL-6 assay kit (431315, BioLegend), TNF-α assay kit (430907, BioLegend), Bicinchoninic acid assay kit (PK10026, Proteintech), Enhanced chemiluminescence detection kit (P0018S, Beyotime Biotechnology), MitoTracker Green (C1996S, Beyotime Biotechnology), MitoSOX Red (S0061S), JC-1 (sc53424, Beyotime Biotechnology), Hoechst 33342 (C1026, Beyotime Biotechnology) and Protein A/G Magnetic Beads (88803, Thermo Fisher Scientific).

### Methods

#### 1. Collection of bovine mammary tissue samples.

Mammary tissue samples were collected from five healthy cows and five cows diagnosed with mastitis at Guangze Ecological Farm in Jilin Province. Cows with mastitis were initially screened based on clinical symptoms, including redness and swelling of the affected udder, somatic cell count in milk >1,000,000 cells/mL, a positive result in the California Mastitis Test, and detection of *S. aureus* as the pathogen. After local anesthesia with 2% lidocaine, mammary tissue samples were surgically excised and placed into 2 mL centrifuge tubes. The samples were rapidly frozen in liquid nitrogen and stored at –80 °C. All tissue specimens were smaller than 1 cm in diameter.

Following surgery, the cows were monitored for one week, and the surgical wounds were regularly inspected to ensure proper healing. Under the supervision of a veterinarian, farm-supplied medications were administered during the first three days post-surgery to prevent infection and alleviate pain. All cows recovered within one week.

#### 2. Bacterial culture.

*S. aureus* strain SA113 (ATCC 35556, American Type Culture Collection, USA) was inoculated into Luria–Bertani broth and cultured at 37 °C for 12–16 h. The bacterial suspension was then collected by centrifugation at 5,000 rpm for 5 minutes, and the pellet was resuspended in sterile phosphate-buffered saline. Bacterial growth was monitored by measuring the optical density at 600 nm to establish a growth curve, and bacterial counts were quantified based on the growth curve.

#### 3. Cell culture and treatments.

Bovine mammary epithelial cells (MAC-T) were cultured in Dulbecco’s modified Eagle’s medium (90%) supplemented with 10% fetal bovine serum (FBS) at 37 °C in a humidified incubator with 5% CO₂. When the cells reached approximately 70% confluence, they were infected with live *S. aureus* by adding bacteria to the culture medium at the indicated MOI and incubating for 6 h. During this period, intracellular and extracellular bacteria were allowed to coexist, and no antibiotic protection or extracellular bacterial killing steps were applied.

For SIRT5 overexpression experiments, cells at ~70% confluence were transfected with a plasmid using Lipofectamine 2000 and cultured for 48 h post-transfection. The overexpression plasmid was constructed by cloning the bovine SIRT5 coding sequence (CDS; GenBank accession no. NM_001034295.2, nucleotides 117–1049) into a pcDNA3.1(+) expression vector under the control of a CMV promoter.

For TLR2 knockdown experiments, cells at ~70% confluence were transfected with a small interfering RNA (siRNA) targeting bovine TLR2, designed based on the bovine TLR2 mRNA sequence (GenBank accession no. NM_174197.2), using Lipofectamine 2000 (Invitrogen) according to the manufacturer’s instructions. After 24–48 h of incubation, cells were used for subsequent functional assays.

For mechanistic experiments examining *S*. *aureus*–induced mitochondrial stress, cells were pretreated with 150 ng/mL Hla, 10 ng/mL TNF-α, or 150 ng/mL Pam3CSK4 for 6 h. After pretreatment, the medium was removed and cells were washed with PBS, followed by infection with live S. aureus at an MOI of 6 for 6 h.

For mitochondrial fission modulation, cells were pretreated with 25 μM Mdivi-1 for 6 h or 10 μM TA9 for 2 h, followed by the indicated treatments.

#### 4. Measurement of oxidative stress indicators.

Commercial assay kits were used to measure MDA, GPx, SOD and GSH/GSSG levels. All procedures were performed according to the manufacturers’ instructions.

#### 5. LDH assay.

Commercial assay kits were used to measure LDH level. All procedures were performed according to the manufacturers’ instructions.

#### 6. Measurement of proinflammatory cytokines.

The levels of IL-1β, IL-6, and TNF-α were determined using ELISA kits, following the manufacturers’protocols.

#### 7. Hematoxylin and eosin staining.

Mammary tissue blocks (~0.5 cm) were thoroughly rinsed and fixed in 4% paraformaldehyde for 24 h. After fixation, the tissues were dehydrated, cleared, embedded in paraffin, and sectioned at a thickness of 5–8 μm. The sections were then baked, deparaffinized, and rehydrated, followed by staining with hematoxylin and eosin (HE). Histological images were acquired using a light microscope.

#### 8. Western Blot.

Total protein was extracted from tissues or cells using NP‑40 lysis buffer, and protein concentration was determined by the Bicinchoninic acid assay. Equal amounts of protein were separated by SDS‑PAGE and transferred onto polyvinylidene difluoride membranes using a wet transfer method. The membranes were blocked with 5% non‑fat milk for 2 h at room temperature and washed with Tris-buffered saline with Tween 20 (TBST). Primary antibodies were incubated overnight at 4 °C, followed by washing with TBST. The membranes were then incubated with HRP‑conjugated goat anti‑rabbit or goat anti‑mouse secondary antibodies at room temperature for 1 hour. After additional washes with TBST, protein bands were visualized using enhanced chemiluminescence, and band intensities were quantified using ImageJ software.

#### 9. Quantitative real‑time PCR (qRT‑PCR).

Total RNA was extracted and subjected to qRT-PCR to determine mRNA expression levels. Primer sequences are listed in Table 1, and the qRT-PCR reaction system is summarized in Table 2.

**Table 1:** Primers.

Gene	Primer
*CXCL10*	Forward: AAG TCA TTC CTG CAA GTC AAT CCT Reverse: TTG ATG GTC TTA GAT TCT GGA TTC AG
*CCL2*	Forward: CGC TCA GCC AGA TGC AAT TA Reverse: GCC TCT GCA TGG AGA TCT TCT T
*IL-8*	Forward: TAGCAAAATTGAGGCCAAGG Reverse: AAACCAAGGCACAGTGGAAC
*SIRT5*	Forward: ACAATGGCTCGTCCAAGTTC Reverse: CCAGTAACCTCCTGCTCCTCT
*β-actin*	Forward: CACCAACTGGGACGACAT Reverse: ATACAGGGACAGCACAGC
*IL-6*	Forward: CTGAAGCAAAAGATCGCAGATCTA Reverse: CTCGTTTGAAGACTGCATCTTCTC
*IL1-β*	Forward: GAGCCTGTCATCTTCGAAACG Reverse: GCACGGGTGCGTCACA
*TNF-α*	Forward: TAACAAGCCAGTAGCCCACG Reverse: GCAAGGGCTCTTGATGGCAGA

**Table 2:** qRT‑PCR reaction system.

Reagent	Volume added
DEPC‑treated water	3μL
cDNA	5μL
Forward primer	1μL
Reverse primer	1μL
SYBR Green master mix	10μL

#### 10. Mitochondrial staining.

After removing the culture medium from treated cells seeded in confocal dishes or 24-well plates, cells were incubated with MitoTracker Green or MitoSOX Red working solution at 37°C in the dark for 10 min. After incubation, the staining solution was removed, and cells were subsequently incubated with Hoechst 33342 working solution at 37°C in the dark for an additional 10 min. After staining, the dye solution was replaced with fresh medium, and mitochondrial signals and nuclei were observed and imaged using a laser scanning confocal microscope or a fluorescence microscope.

#### 11. JC-1 staining.

JC-1 staining was performed using a commercial kit according to the manufacturer’s instructions. After staining, mitochondrial membrane potential was observed and imaged under a laser scanning confocal microscope.

#### 12. Co-IP.

After total cellular protein was extracted, it was incubated overnight at 4 °C with a primary antibody specific to the target protein to form an immune complex. The next day, pre-washed protein A/G magnetic beads were added and incubated at room temperature for 1 h to facilitate binding between the antigen–antibody complex and the beads. After incubation, the beads were washed multiple times and then resuspended in 1 × sodium dodecyl sulfate loading buffer to elute the bound proteins. After centrifugation to remove the beads, the supernatant containing the immunoprecipitated proteins was collected and subjected to subsequent Western blot analysis.

#### 13. Intracellular bacterial load.

During the final 1 h of *S. aureus* infection, the culture medium was replaced with DMEM containing 100 μg/mL gentamicin. At the end of infection, cells were washed three times with PBS and then lysed in 150 μL of 0.25% Triton X-100 and 150 μL of 0.25% trypsin. The cell lysates were diluted 1:1000, plated on LB agar, and incubated at 37°C for 24 h, after which colony-forming units were counted.

#### 14. Liquid chromatography–tandem mass spectrometry (LC–MS/MS).

SIRT5 immunoprecipitation samples were separated by SDS-PAGE and stained with Coomassie Brilliant Blue for 2 h, followed by destaining until the background was clear. The target bands were excised from the gel and subjected to in-gel trypsin digestion: sequential destaining, reduction with DTT, alkylation with IAA, and overnight digestion with trypsin (10 ng/μL) at 37 °C. Peptides were extracted using 50% acetonitrile/5% formic acid and 100% acetonitrile, vacuum-dried, and resuspended in 0.1% formic acid/2% acetonitrile.

Peptides were separated by liquid chromatography on a homemade C18 column (25 cm × 75 μm) using an EASY-nLC 1200 system and analyzed by tandem mass spectrometry (LC–MS/MS) on a Q Exactive HF-X mass spectrometer (Thermo Fisher Scientific). The full MS resolution was set to 60,000, the HCD collision energy to 28%, and the MS/MS resolution to 30,000. Raw MS data were analyzed using Proteome Discoverer v2.4 with the Bos_taurus_9913_PR_20241023.fasta database. Trypsin/P was specified as the cleavage enzyme, carbamidomethylation (C) was set as a fixed modification, oxidation (M) and N-terminal acetylation were set as variable modifications, with up to two missed cleavages allowed. The mass tolerance was set to 10 ppm for precursor ions and 0.02 Da for fragment ions, and the false discovery rate (FDR) was controlled below 1%.

#### 15. Data analysis.

All statistical parameters were calculated using GraphPad Prism 8 and Excel software. Differences were analyzed using t-tests (for two groups) and one-way ANOVA for multiple comparisons. *p* < 0.05 was considered statistically significant. Values are expressed as mean ± standard error of the mean (SEM).

## Supporting information

S1 Fig Graphical Abstract. *Staphylococcus aureus* (*S. aureus*) infection in bovine mammary tissue elicits pronounced oxidative stress, which subsequently induces senescence in mammary epithelial cells. During infection, the *S. aureus* toxin α-hemolysin (Hla) first triggers an intracellular inflammatory response and then provokes mitochondrial stress. In this process, the interaction between Sirtuin 5 (SIRT5) and the ubiquitin-conjugating enzyme ubiquitin C (UBC) is enhanced, thereby promoting SIRT5 ubiquitination and degradation and leading to a marked reduction in its protein level. Because SIRT5 functions as a desuccinylase, its downregulation increases the succinylation of the mitochondrial fission protein dynamin-related protein 1 (DRP1) and suppresses its ubiquitin-mediated degradation, which in turn drives excessive mitochondrial fragmentation and mitochondrial stress, exacerbates global cellular oxidative stress, and accelerates cellular senescence.
(DOCX)

S2 Fig Effect of Lactate Treatment on Mitochondrial Stress in Bovine Mammary Epithelial Cells. (a) Mitochondria were labeled with MitoTracker Green, and nuclei were stained with Hoechst in Lactate-treated bovine mammary epithelial cells. A laser confocal microscope was used for observation (n = 3). Scale bar = 5 μm. (b) Lactate treatment was applied to bovine mammary epithelial cells, which were then stained using a JC-1 commercial kit. (c) Mitochondrial superoxide was detected using MitoSOX Red in Lactate-treated cells, and nuclei were counterstained with Hoechst. Fluorescence images were acquired using a laser scanning confocal microscope (n = 3). Scale bar = 20 μm.
(DOCX)

S3 Fig Verification of SIRT5 overexpression at the mRNA level. Relative SIRT5 mRNA expression in bovine mammary epithelial cells transfected with the SIRT5 overexpression plasmid, as determined by qRT-PCR. Data are presented as mean ± SD (n = 3). P values are indicated in the figure.
(DOCX)
