# Supplementary material for: The UBC/SIRT5/DRP1 axis regulates mitochondrial dynamics to alleviate Staphylococcus aureus-induced oxidative stress and senescence in bovine mammary epithelial cells
Source: PLoS Pathog. 2026 Feb 12;22(2):e1013975. doi: 10.1371/journal.ppat.1013975 (PMC12919931; doi:10.1371/journal.ppat.1013975)
Supplement: S1 Fig — Staphylococcus aureus (S. aureus) infection in bovine mammary tissue elicits pronounced oxidative stress, which subsequently induces senescence in mammary epithelial cells. During infection, the S. aureus toxin α-hemolysin (Hla) first triggers an intracellular inflammatory response and then provokes mitochondrial stress. In this process, the interaction between Sirtuin 5 (SIRT5) and the ubiquitin-conjugating enzyme ubiquitin C (UBC) is enhanced, thereby promoting SIRT5 ubiquitination and degradation and leading to a marked reduction in its protein level. Because SIRT5 functions as a desuccinylase, its downregulation increases the succinylation of the mitochondrial fission protein dynamin-related protein 1 (DRP1) and suppresses its ubiquitin-mediated degradation, which in turn drives excessive mitochondrial fragmentation and mitochondrial stress, exacerbates global cellular oxidative stress, and accelerates cellular senescence. (DOCX) [file ppat.1013975.s001.docx]

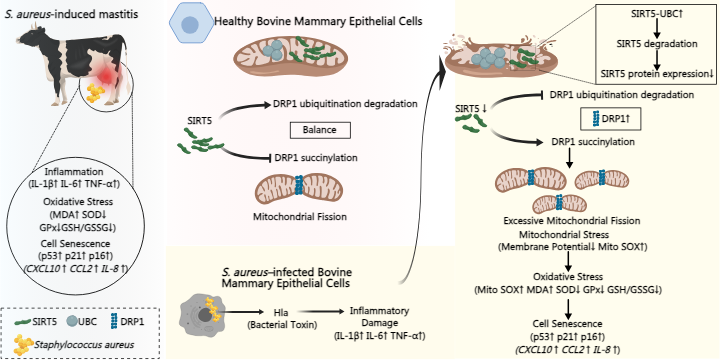


**S1 Fig. Graphical Abstract.** *Staphylococcus aureus* (*S. aureus*) infection in bovine mammary tissue elicits pronounced oxidative stress, which subsequently induces senescence in mammary epithelial cells. During infection, the *S. aureus* toxin α-hemolysin (Hla) first triggers an intracellular inflammatory response and then provokes mitochondrial stress. In this process, the interaction between Sirtuin 5 (SIRT5) and the ubiquitin-conjugating enzyme ubiquitin C (UBC) is enhanced, thereby promoting SIRT5 ubiquitination and degradation and leading to a marked reduction in its protein level. Because SIRT5 functions as a desuccinylase, its downregulation increases the succinylation of the mitochondrial fission protein dynamin-related protein 1 (DRP1) and suppresses its ubiquitin-mediated degradation, which in turn drives excessive mitochondrial fragmentation and mitochondrial stress, exacerbates global cellular oxidative stress, and accelerates cellular senescence.
