# Supplementary material for: The UBC/SIRT5/DRP1 axis regulates mitochondrial dynamics to alleviate Staphylococcus aureus-induced oxidative stress and senescence in bovine mammary epithelial cells
Source: PLoS Pathog. 2026 Feb 12;22(2):e1013975. doi: 10.1371/journal.ppat.1013975 (PMC12919931; doi:10.1371/journal.ppat.1013975)
Supplement: S2 Fig — (a) Mitochondria were labeled with MitoTracker Green, and nuclei were stained with Hoechst in Lactate-treated bovine mammary epithelial cells. A laser confocal microscope was used for observation (n = 3). Scale bar = 5 μm. (b) Lactate treatment was applied to bovine mammary epithelial cells, which were then stained using a JC-1 commercial kit. (c) Mitochondrial superoxide was detected using MitoSOX Red in Lactate-treated cells, and nuclei were counterstained with Hoechst. Fluorescence images were acquired using a laser scanning confocal microscope (n = 3). Scale bar = 20 μm. (DOCX) [file ppat.1013975.s002.docx]

**
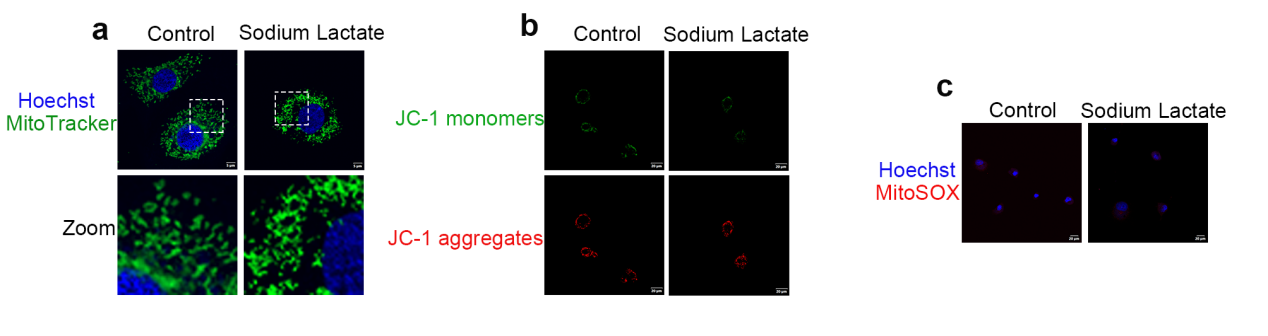
**

**S2 Fig. Effect of Lactate Treatment on Mitochondrial Stress in Bovine Mammary Epithelial Cells**

(a) Mitochondria were labeled with MitoTracker Green, and nuclei were stained with Hoechst in Lactate-treated bovine mammary epithelial cells. A laser confocal microscope was used for observation (n = 3). Scale bar = 5 μm. (b) Lactate treatment was applied to bovine mammary epithelial cells, which were then stained using a JC-1 commercial kit. (c) Mitochondrial superoxide was detected using MitoSOX Red in Lactate-treated cells, and nuclei were counterstained with Hoechst. Fluorescence images were acquired using a laser scanning confocal microscope (n = 3). Scale bar = 20 μm.
