# Supplementary material for: The UBC/SIRT5/DRP1 axis regulates mitochondrial dynamics to alleviate Staphylococcus aureus-induced oxidative stress and senescence in bovine mammary epithelial cells
Source: PLoS Pathog. 2026 Feb 12;22(2):e1013975. doi: 10.1371/journal.ppat.1013975 (PMC12919931; doi:10.1371/journal.ppat.1013975)
Supplement: S3 Fig — Relative SIRT5 mRNA expression in bovine mammary epithelial cells transfected with the SIRT5 overexpression plasmid, as determined by qRT-PCR. Data are presented as mean ± SD (n = 3). P values are indicated in the figure. (DOCX) [file ppat.1013975.s003.docx]

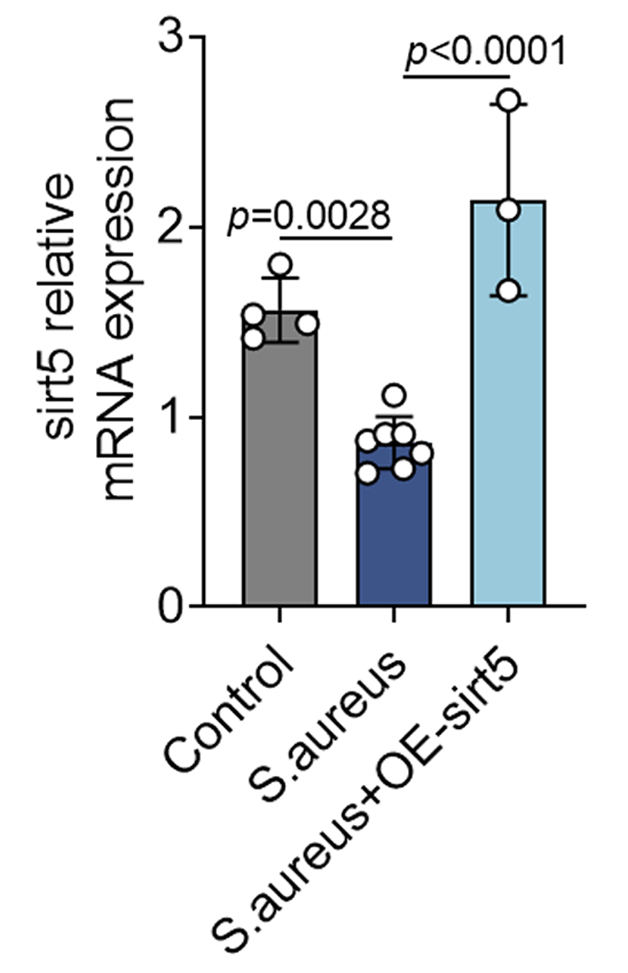


**S3 Fig. Verification of SIRT5 overexpression at the mRNA level.**

Relative SIRT5 mRNA expression in bovine mammary epithelial cells transfected with the SIRT5 overexpression plasmid, as determined by qRT-PCR. Data are presented as mean ± SD (n = 3). *p* values are indicated in the figure.
